# Administration of nicotinamide mononucleotide improves oocyte quality of obese mice

**DOI:** 10.1111/cpr.13303

**Published:** 2022-07-10

**Authors:** Luyao Wang, Yurong Chen, Jiarui Wei, Fucheng Guo, Leyi Li, Zhen Han, Zhengzhu Wang, Haibo Zhu, Xiaoling Zhang, Ziyi Li, Xiangpeng Dai

**Affiliations:** ^1^ Key Laboratory of Organ Regeneration and Transplantation of Ministry of Education, First Hospital Jilin University Changchun China; ^2^ National‐Local Joint Engineering Laboratory of Animal Models for Human Disease, First Hospital Jilin University Changchun China; ^3^ Center of Reproductive Medicine & Center of Prenatal Diagnosis, First Hospital Jilin University Changchun China

## Abstract

**Objectives:**

Obesity has become a common health concern around the world. Maternal obesity could cause poor reproductive outcomes due to chronic ovarian inflammation and decreased oocyte quality. However, the strategies to improve the poor reproductive outcomes of obese females have not been fully studied. In this study, we aimed to explore the effects and underlying mechanisms of nicotinamide mononucleotide (NMN) on oocyte quality and reproductive performance of obese mice.

**Materials and Methods:**

The obese mouse model was established by feeding high‐fat diet which was confirmed by body weight record, fasting blood glucose test and oral glucose tolerance test. The expression of ovary development related genes and inflammation related genes, including Lhx8, Bmp4, Adgre1, Ccl2, TNF‐α, Gal‐3, Clec10a and IL‐10 in ovaries and the expression of Bax and Sod1 in oocytes were detected using quantitative reverse transcription PCR (RT‐qPCR). The adipose size of abdominal fat tissue was determined with haematoxylin and eosin (H&E) staining. Immunofluorescence staining was performed to measure the ROS level, spindle/chromosome structure, mitochondrial function, actin dynamics and DNA damage of oocytes.

**Results:**

The administration of NMN restored ovarian weight and reduced the adipose size of abdominal fat tissue and ovarian inflammation in high fat diet (HFD) mice. Furthermore, NMN treatment improved the oocytes quality partially by restoring the mitochondrial function and actin dynamics, reducing meiotic defects, DNA damage and ROS level and lipid droplet distribution of oocytes in HFD mice. On the long‐term effect, NMN restored offspring body weight of HFD mice.

**Conclusion:**

NMN could improve the oocyte quality of HFD‐induced obese mice.

## Graphical abstract

Diagram illustrating the beneficial effects of NMN on the productivity of mice especially the ovary and oocyte quality in obese female mice. Intraperitoneal injection of NMN improved ovary quality of high fat diet mice. The administration of NMN restored the mitochondrial function and actin dynamics and reduced meiotic defects, DNA damage and ROS level of oocytes in obese mice. The administration of NMN also restored offspring body weight of obese mice. Therefore, the productivity of obese mice could be improved after NMN supplement.

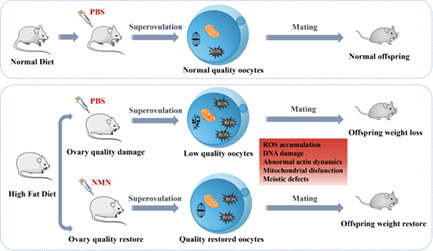

## 1 INTRODUCTION

Obesity has become a major global health issue with the rise of overweight.^1^ Maternal obesity is closely related to poor reproductive outcomes, including early pregnancy failure, preeclampsia and neonatal conditions.^2^, ^3^ Chronic inflammation caused by obesity impacts ovarian physiology and impairs reproductive health.^4^ Multiple studies have found that the oocyte quality of obese mice is impaired, due to mitochondrial dysfunction, meiotic abnormalities and oxidative stress caused by obesity.^5^ The oocyte mitochondrial dysfunction of obese mice can be transmitted to offspring, increasing the risk of insulin resistance in offspring.^6^ The adverse effects of maternal obesity on oocytes and preimplantation embryos may lead to long‐term metabolic diseases in offspring.^7^, ^8^ Thus, low‐quality of oocytes is the main reason for the poor reproductive outcomes of obese women.^5^ However, the therapeutic strategies to improve oocyte quality of obese women have not been well explored.

Nicotinamide adenine dinucleotide (NAD^+^) is an important cofactor which participates in various biological processes, including energy metabolism, DNA repair, genomic stability and cell survival.^9^, ^10^ The NAD^+^ level decreased with age and metabolic abnormalities, such as obesity and diabetes.^11^ Study has shown that the loss of NAD^+^ decreased oocyte quality and compromised early embryonic development in obese mice.^12^ Nicotinamide mononucleotide (NMN), a key precursor of NAD^+^, can enhance NAD^+^ biosynthesis and reverse defects caused by insufficient NAD^+^. Therefore, administration of NMN can alleviate physiological abnormality in ageing mice.^13^ NMN can also benefit the patients of type 2 diabetes induced by high‐fat diet and enhance liver insulin sensitivity.^11^ In 2020, Miao et al. and Bertoldo et al. separately reported that NMN supplementation recovered NAD^+^ level and improved the oocyte quality in ageing mice.^14^, ^15^ Despite progress has been made in the application of NMN towards ageing and obesity, the effect of NMN on oocyte quality and female fertility in obese mice has not been fully explored.

In our study, we established obesity model by high‐fat diet feeding, and further investigated the effect of NMN on oocyte quality and reproductive performance of obese mice. Our results showed that the in vivo administration of NMN reduces ovarian inflammation, improves oocyte quality and restores offspring weight of obese female mice. Furthermore, the NMN supplementation improved oocyte quality of obese mice by recovering mitochondrial function, reducing the accumulated ROS and suppressing apoptosis.

## 2 MATERIALS AND METHODS

### 2.1 Mice

Three‐week‐old female C57BL/6J mice and 11‐week‐old male C57BL/6J mice were purchased from Charles River Laboratory (Beijing, China). Mice were housed in specific pathogen‐free (SPF) conditions on a 12‐h light:12‐h dark cycle at constant temperature and under controlled humidity at the First Hospital of Jilin University. The female mice were randomly divided to two groups at the age of 4 weeks. One group received a normal diet (Cat#: XTCON50J, Jiangsu Xietong, Nanjing, China) and the other group received a high fat diet (Cat#: XTHF60, Jiangsu Xietong, Nanjing, China) for 12 weeks. All research procedures followed the guidelines of Institutional Animal Care and Use Committee of the First Hospital of Jilin University. Sixteen‐week‐old obese mice were intraperitoneally injected with NMN (Selleck) at the dose of 200 mg/kg/day or the equivalent volume of PBS for 10 consecutive days. The dosage concentration and dosing days are determined based on previous study.^14^ A single 12‐week‐old male C57BL/6J mouse with proven fertility was caged overnight with a single female mouse for mating. Vaginal plugs in the female mice were detected in the next morning. The numbers and weights of offspring were recorded on the day of birth.

### 2.2 Oocyte collection

Female C57BL/6J mice (16 weeks old) were superovulated with 7.5 IU pregnant mare serum gonadotropin (PMSG) (Ningbo Second Hormone Factory, China) followed by 7.5 IU human chorionic gonadotropin (hCG) (Ningbo Second Hormone Factory, China) 48 h later. The cumulus‐oocyte complexes were collected from the oviducts 13–16 h after hCG injection and cumulus cells were removed by 1 mg/ml hyaluronidase (Sigma) incubation.

### 2.3 Fasting blood glucose and oral glucose tolerance test

For fasting blood glucose, the blood glucose level of mice was measured after they were fasted for 12 h. For oral glucose tolerance test (OGTT), mice were fasted for 4 h followed by a glucose administration (2 g/kg) by gavage, then the blood glucose level was measured at 0, 30, 60 and 120 min after the glucose load. The blood glucose was detected with one Touch Ultra glucose meter (ACCU‐CHEK Performa, Roche Diabetes Care GmbH).

### 2.4 RNA isolation and quantitative real‐time polymerase chain reaction

Total RNA was exacted from 50 oocytes and reversed to cDNA using SuperScript™ IV CellsDirect™ cDNA Synthesis Kit (Invitrogen). The total RNA of ovary was extracted using TRIzol (Takara, Japan) and reversed to cDNA using RevertAid Master Mix (https://www.thermofisher.cn/document-connect/document-connect.html?url=https://assets.thermofisher.cn/TFS-Assets/LSG/manuals/MAN0018936_RevertAidMasterMix_UG.pdf; Thermo Fisher Scientific). Real‐time polymerase chain reaction (RT‐PCR) was conducted using PowerUp SYBR Green Master Mix (https://www.thermofisher.cn/document-connect/document-connect.html?url=https://assets.thermofisher.cn/TFS-Assets/LSG/manuals/100031508_PowerUp_SYBRgreen_QRC.pdf; Applied Biosystems). The primers used for quantitative RT PCR were listed in Table 1.

**TABLE 1:** Primer sequences of genes for quantitative real‐time PCR

Gene	Primer sequence
Lhx8	Forward, 5′‐ACACGAGCTGCTACATTAAGGA‐3′
	Reverse, 5′‐CCAGTCAGTCGAGTGGATGTG‐3′
Bmp4	Forward, 5′‐TTGATACCTGAGACCGGGAAG‐3′
	Reverse, 5′‐ACATCTGTAGAAGTGTCGCCTC‐3′
Adgre1	Forward, 5′‐CTGCACCTGTAAACGAGGCTT‐3′
	Reverse, 5′‐GCAGACTGAGTTAGGACCACAA‐3′
Ccl2	Forward, 5′‐TAAAAACCTGGATCGGAACCAAA‐3′
	Reverse, 5′‐GCATTAGCTTCAGATTTACGGGT‐3′
TNF‐α	Forward, 5′‐CCAGACCCTCACACTCAGATC‐3′
	Reverse, 5′‐CACTTGGTGGTTTTGCTACGAC‐3′
Gal‐3	Forward, 5′‐TCCTGGAGGCTATCCTGCTG‐3′
	Reverse, 5′‐TGTTTGCGTTGGGTTTCACTG‐3′
Clec10a	Forward, 5′‐CAATGTGGTTAGTTGGATCGGC‐3′
	Reverse, 5′‐CCCAGTTCTTAAAGCCTTTCTCA‐3′
IL‐10	Forward, 5′‐TTAATAAGCTCCAAGACCAAGG‐3′
	Reverse, 5′‐CATCATGTATGCTTCTATGCAG‐3′
Bax	Forward, 5′‐TGAAGACAGGGGCCTTTTTG‐3′
	Reverse, 5′‐AATTCGCCGGAGACACTCG‐3′
Sod1	Forward, 5′‐AACCAGTTGTGTTGTCAGGAC‐3′
	Reverse, 5′‐CCACCATGTTTCTTAGAGTGAGG‐3′
Actin	Forward, 5′‐TTCAACACCCCAGCCATG‐3′
	Reverse, 5′‐CCTCGTAGATGGGCACAGT‐3′

### 2.5 Histological analysis of fat

Mouse abdominal fat used for histological analysis was collected and fixed in 10% neutral formalin buffered solution for at least 24 h, dehydrated, and embedded in paraffin. Paraffin‐embedded fat was then sectioned at a thickness of 5 μm for further haematoxylin and eosin (H&E) staining.

### 2.6 Immunofluorescence staining

Indirect immunofluorescence was performed as previously described with some modifications.^16^ The zona pellucida of oocytes was removed using acidic tyrode solution (Sigma) and oocytes were then fixed with 4% paraformaldehyde. After three washes in phosphate‐buffered saline (PBS) with 0.1% polyvinylpyrrolidone (PVP), oocytes were permeabilized with 1% Triton X‐100 for 20 min at room temperature. After washing 3 times with PBS containing 0.1% PVP, oocytes were blocked with PBS containing 2% bovine serum albumin (BSA) for 1 h. Then oocytes were incubated with anti‐tubulin antibody (1:100) or anti‐γ‐H2A.X antibody (1:100) at 4°C overnight. After washing 3 times, oocytes were incubated with secondary antibodies for 1 h at 37°C. Then oocytes were counterstained with Hoechst 33342 (10 μg/ml). Finally, oocytes were transferred to slides and mounted using prolong Gold Antifade Mountant (Invitrogen) and observed on a Zeiss LSM880 confocal microscope.

For active mitochondrion staining, oocytes were stained with 500 nM MitoTracker Red CMXRos (Thermo Fisher Scientific) for 30 min at 37°C. For mitochondrial membrane potential assessment, oocytes were stained with 2 μM MitoProbe JC‐1 (Beyotime Biotechnology, China) at 37°C. For ROS staining, oocytes were treated with the Reactive Oxygen Species Assay Kit (Beyotime Biotechnology, China) for 30 min at 37°C. For actin cytoskeleton staining, oocytes were incubated in with Actin‐Tracker Red (Beyotime Biotechnology, China) for 30 min at 37°C. For lipid drop staining, oocytes were fixed with 4% paraformaldehyde overnight at 4°C. After washing 3 times with PBS containing 0.1% PVP, the oocytes were incubated with 4,4‐difluoro‐4‐bora‐3a,4a‐diaza‐s‐indacene 493/503 (Thermo Fisher Scientific) for 30 min at 37°C.

### 2.7 Statistical analysis

All values from at least three independent replicates were expressed as mean ± SEM. Data were analysed by *t*‐test or chi‐squared test using GraphPad Prism 7 statistical software. *P* < 0.05 was considered as significant.

## 3 RESULTS

### 3.1 Establishment of obese mouse model

**FIGURE 1:**
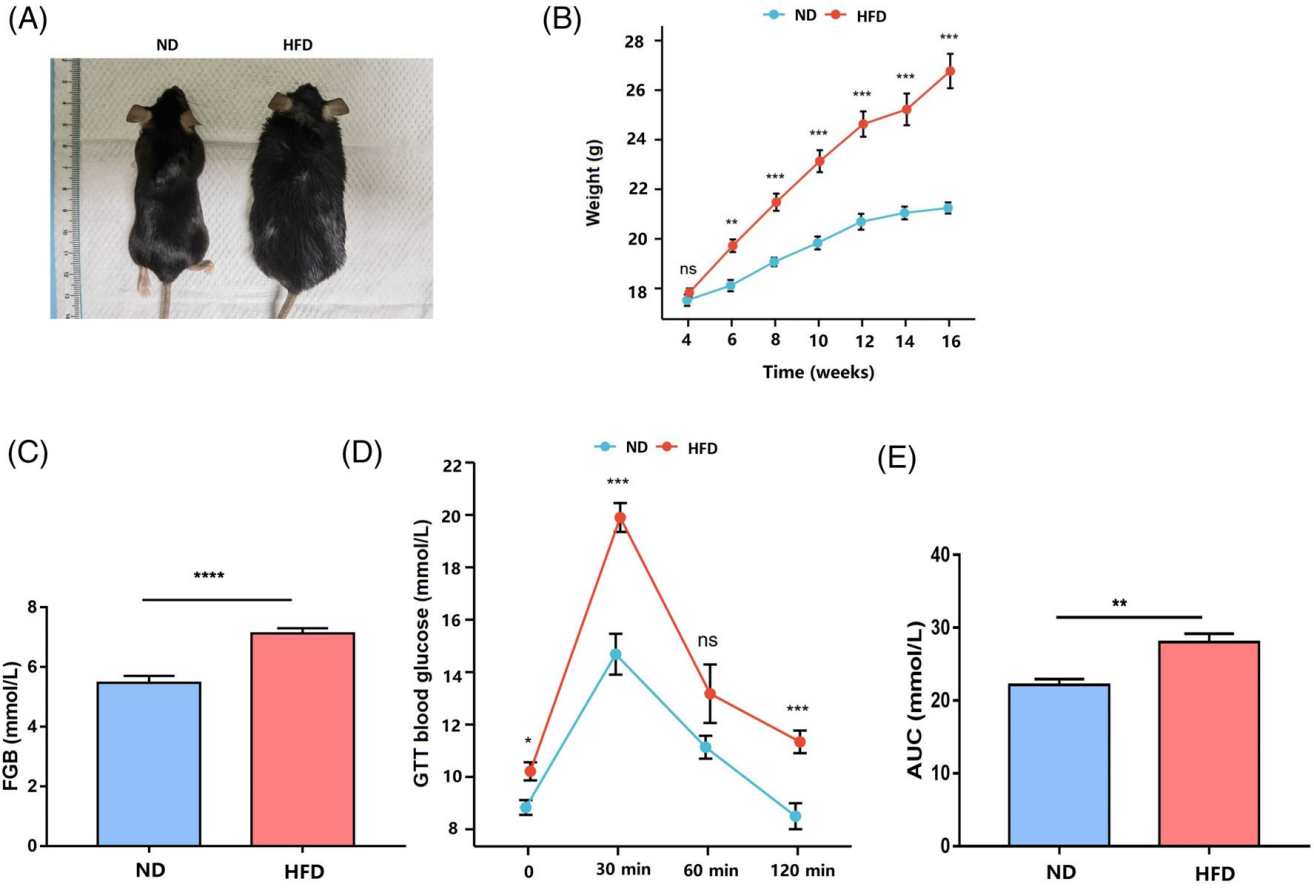
The obese mice model induced by high fat diet. (A) Image of mice after 12‐week feeding. ND, normal diet; HFD, high fat diet. (B) The dynamic body weight of mice fed with high fat diet (*n* = 27) and normal diet (*n* = 9). (C) Fasting blood glucose of normal diet mice (*n* = 34) and high fat diet mice (*n* = 9). (D) Blood glucose of normal diet mice (*n* = 6) and high fat diet mice (*n* = 8) during oral glucose tolerance test. (E) Area under the curve (AUC) of oral glucose tolerance test for normal diet mice (*n* = 6) and high fat diet mice (*n* = 8). *P < 0.05; **P < 0.01; ***P < 0.001; ****P < 0.0001

To establish the obese mouse model, 4‐week‐old mice were fed with normal diet (ND) or high fat diet (HFD) for 12 weeks and the body weight of mice was measured every 2 weeks. The body weight of mice fed with high fat diet (HFD mice) is significantly higher than that of mice fed with normal diet (ND mice) (Figure 1A,B). Consistently, we found the FGB value of HFD group is higher than that of ND group (Figure 1C). Furthermore, the OGTT results indicated that the HFD mice are glucose intolerant (Figure 1D,E). These results indicated that the obese mice model is established successfully.

### 3.2 NMN protected against abnormal metabolism in HFD mice

**FIGURE 2:**
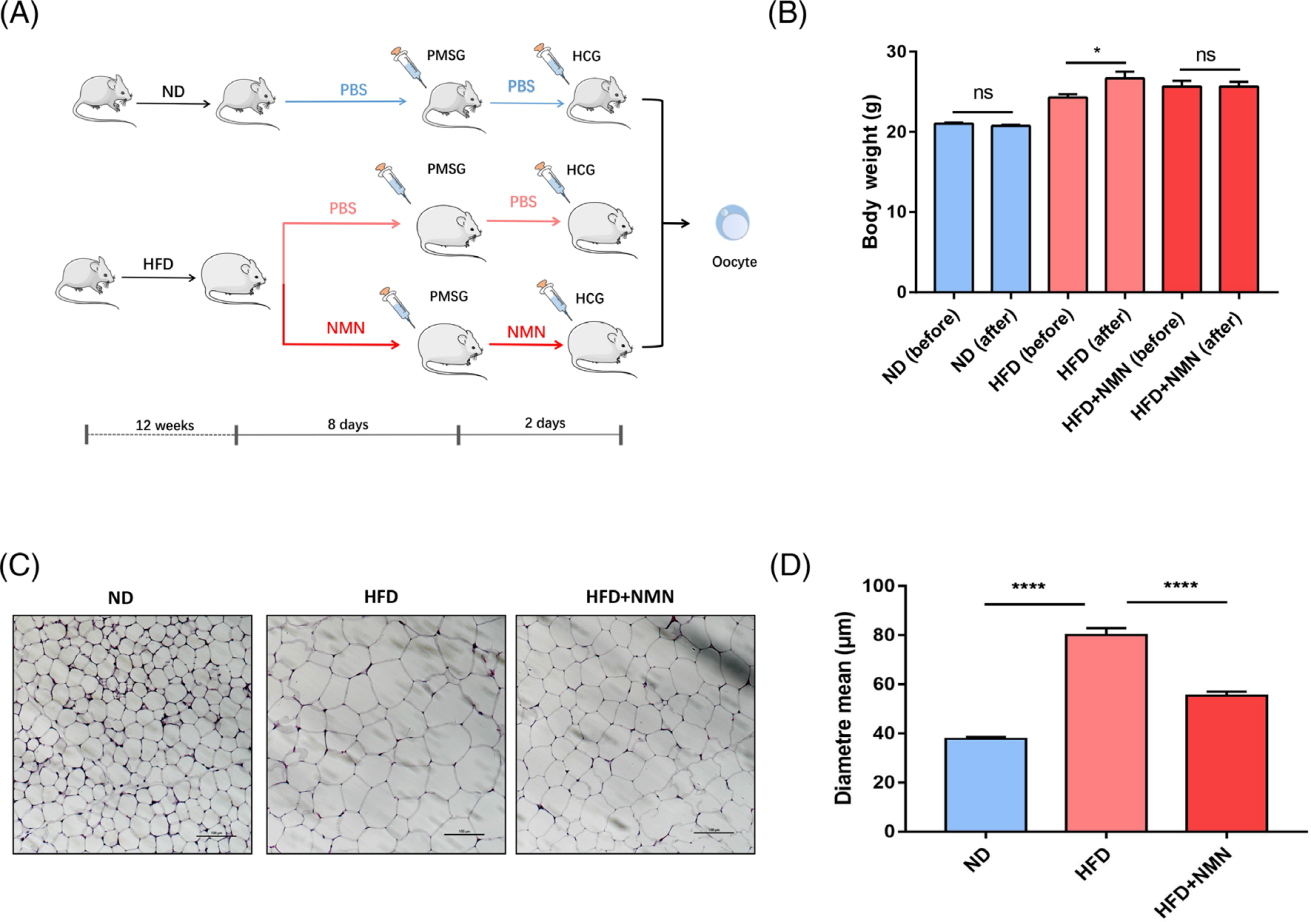
Effect of NMN on body weight and fat size. (A) The schematic of NMN administration and superovulation performed on the indicated mice. The mice in the ND group were fed normal diet and had intraperitoneal administrations of PBS (blue). The mice in the HFD group were fed high‐fat diet and divided to two groups. One group had intraperitoneal administrations of PBS (pink), and the other group had intraperitoneal administrations of NMN (red). (B) The body weight of mice before and after NMN or PBS administrations. ND, normal diet. HFD, high fat diet. HFD + NMN, high fat diet with administrations of NMN. ND, *n* = 5; HFD, *n* = 9, HFD + NMN, *n* = 10. (C) Representative images of fat tissue from ND group, HFD group and HFD + NMN group. Scale bar, 100 μm. HFD + NMN, high fat diet with administrations of NMN. (D) Analysis of adipocyte size in ND group, HFD group and HFD + NMN group. *P < 0.05; ****P < 0.0001

After 12 weeks of dietary intervention, the ND mice and HFD mice were intraperitoneally injected with PBS or NMN for 10 consecutive days (Figure 2A). The body weights of HFD mice treated with PBS were gradually increased, but there was no dramatic change on the body weights of HFD mice treated with NMN (Figure 2B). The adipose size of abdominal fat tissue was significantly larger in HFD mice than in ND mice (Figure 2C), however, NMN administration reduced the adipose size of HFD mice (Figure 2D). These results showed that the administration of NMN protected against abnormal metabolism in HFD mice.

### 3.3 NMN improved the ovary quality of HFD mice

**FIGURE 3:**
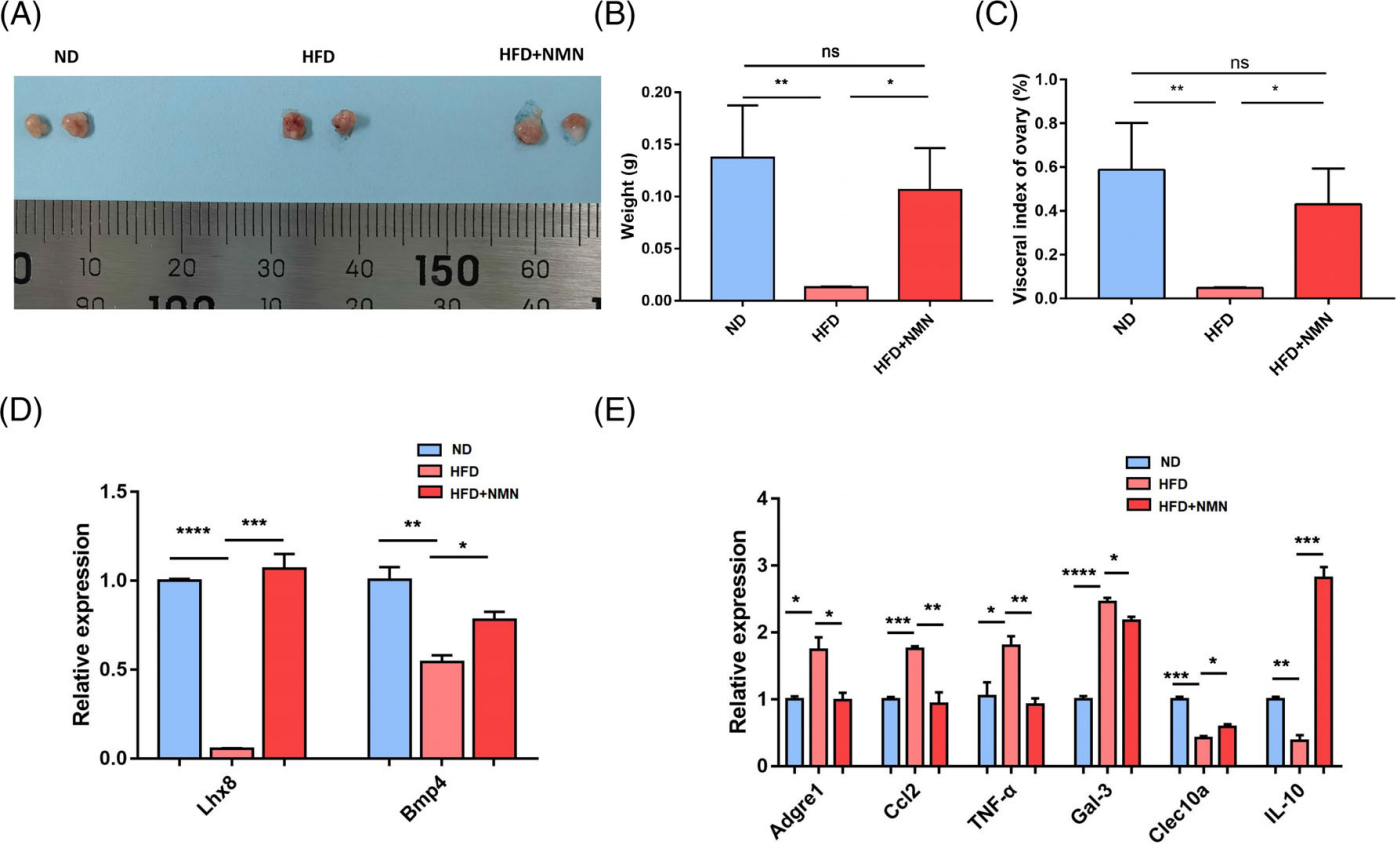
Effect of NMN on ovary quality. (A) Representative images of ovaries from ND group, HFD group and HFD + NMN group. (B) Weight of ovaries collected from ND group, HFD group and HFD + NMN group. (C) Visceral index of ovaries collected from ND group, HFD group and HFD + NMN group. (D) mRNA levels of Lhx8 and Bmp4 were measured by RT‐PCR in ND, HFD and HFD + NMN mice ovaries. (E) mRNA levels of Adgre1, Ccl2, TNF‐α, Gal‐3, Clec10a and IL‐10 were measured by RT‐PCR in ND, HFD and HFD + NMN mice ovaries. *P < 0.05; **P < 0.01; ***P < 0.001; ****P < 0.0001

Next, we sought to investigate the effect of NMN on the ovary physiology of obese female mice. There was no obvious morphological difference on the ovaries among the ND, HFD and HFD + NMN groups (Figure 3A). However, the ovarian weight and ovarian organ index of mice in the HFD group were significantly lower than those in the ND and HFD + NMN group (Figure 3B,C). Given the dramatic difference on ovary weight and ovarian organ index, we further examined the mRNA levels of Bmp4 and Lhx8 which are related to ovary follicular development. The mRNA levels of Bmp4 and Lhx8 in the HFD ovary were decreased while the NMN treatment significantly elevated the mRNA levels of Bmp4 and Lhx8 (Figure 3D). With regard to ovarian inflammation, we examined the mRNA levels of Adgre1, Ccl2, TNF‐α, Gal‐3, Clec10a and IL‐10. The results showed that high fat diet upregulated the mRNA levels of Adgre1, Ccl2, TNF‐α and Gal‐3, while the NMN treatment dramatically decreased their mRNA levels (Figure 3E). However, the mRNA levels of Clec10a and IL‐10 were significantly lower in HFD mice than that in ND mice and HFD + NMN mice (Figure 3E). These results showed that NMN could improve the ovary quality in HFD mice by regulating the expression of critical genes related to ovarian follicle development and inflammation of HFD mice. The raw RT‐PCR data were listed in Supplementary Table 1.

### 3.4 NMN reduced meiotic defects and DNA damage in oocytes of HFD mice

**FIGURE 4:**
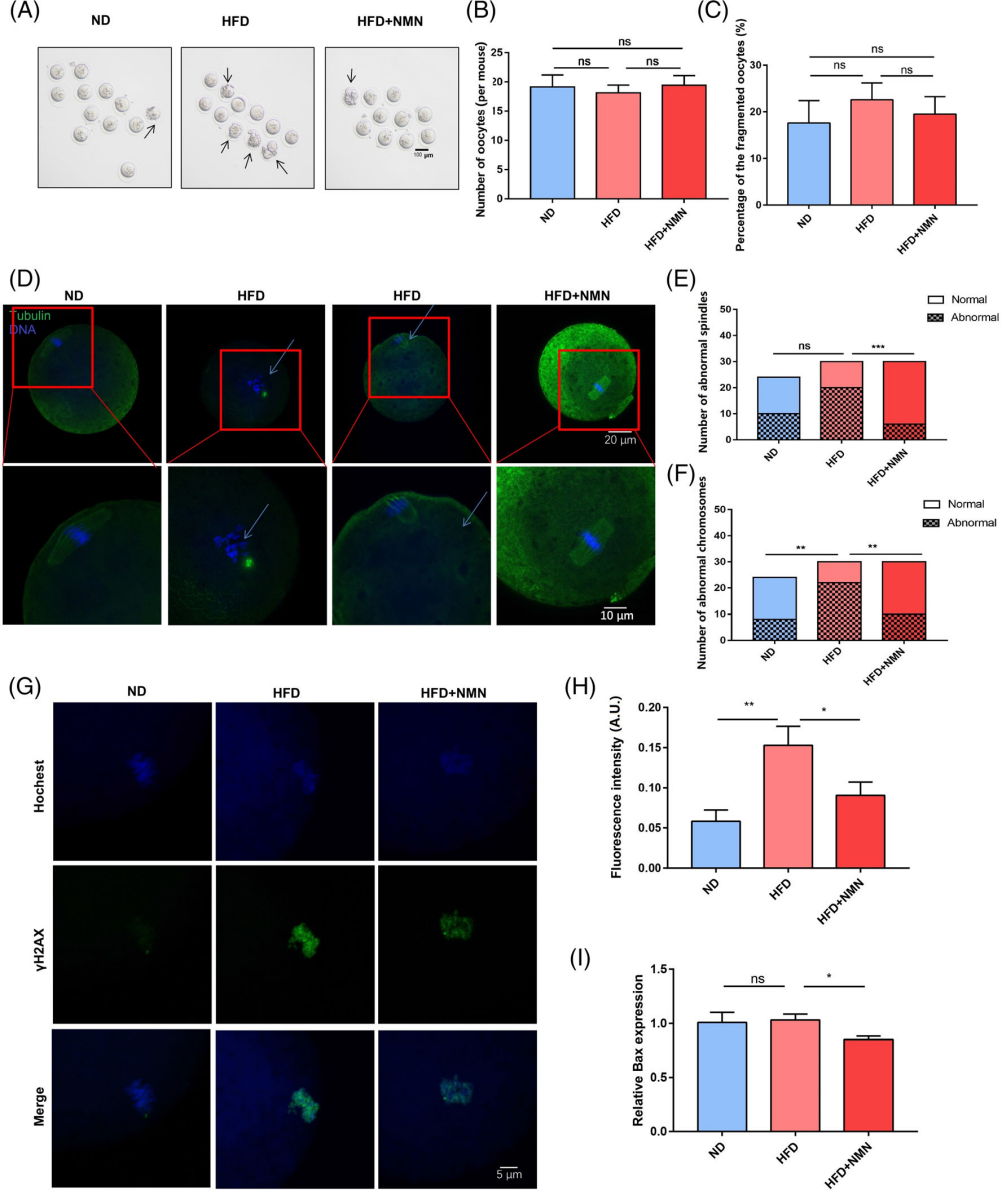
Effect of NMN on spindle‐chromosome structure and DNA damage. (A) Representative images of oocytes collected from ND, HFD and HFD + NMN mice. The arrow points to the abnormal spindle or chromosome. Scale bar, 100 μm. (B) Number of oocytes collected from ND, HFD and HFD + NMN mice. (C) Percentage of the fragmented oocytes collected from ND, HFD and HFD + NMN mice. (D) Representative images of the spindle morphology and chromosome alignment at metaphase II oocytes collected from ND, HFD and HFD + NMN mice. Scale bars, 20 μm, 10 μm. (E) The percentage of aberrant spindles at metaphase II oocytes collected from ND, HFD and HFD + NMN mice. (F) The percentage of misaligned chromosomes at metaphase II oocytes collected from ND, HFD and HFD + NMN mice. (G) Representative images of γH2A.X signals in oocytes collected from ND, HFD and HFD + NMN mice. Scale bar, 5 μm. (H) The fluorescence intensity of γH2A.X signals was quantified in oocytes collected from ND, HFD and HFD + NMN mice. (I) mRNA level of Bax was measured by RT‐PCR in ND, HFD and HFD + NMN mice ovaries. *P < 0.05; **P < 0.01; ***P < 0.001

Moreover, we examined the effect of NMN on oocyte quality in HFD mice. The MII oocytes were isolated from ND mice, HFD mice and HFD + NMN mice (Figure 4A). There was no significant difference on the number of oocytes between three groups (Figure 4B). Although some HFD mice have more fragmented oocytes than ND mice, there was no significant difference on the percentage of fragmented oocytes between the three groups (Figure 4C). Given that the spindle defects and chromosomal abnormalities may frequently occur in MII oocytes of obese mice,^17^ the spindle defects and chromosome misalignment in three groups was further examined. The results showed that NMN supplement could reduce the high frequency of spindle defects and chromosome misalignment induced by HFD (Figure 4D–F). We further evaluated DNA damage in terms of γH2A.X. The results indicated that γH2A.X signals were higher in HFD oocytes than that in ND oocytes, and supplementation of NMN significantly reduced the γH2A.X signals in HFD oocytes (Figure 4G,H). In addition, the qRT‐PCR results showed that the mRNA level of apoptotic factor Bax is lower in HFD + NMN group than that in ND group and HFD group (Figure 4I). These results indicated that the NMN could rescued the DNA damage induced by HFD through reducing the expression of Bax.

### 3.5 NMN reversed the abnormality of ROS level, mitochondrial function and actin dynamics in the oocytes of HFD mice

**FIGURE 5:**
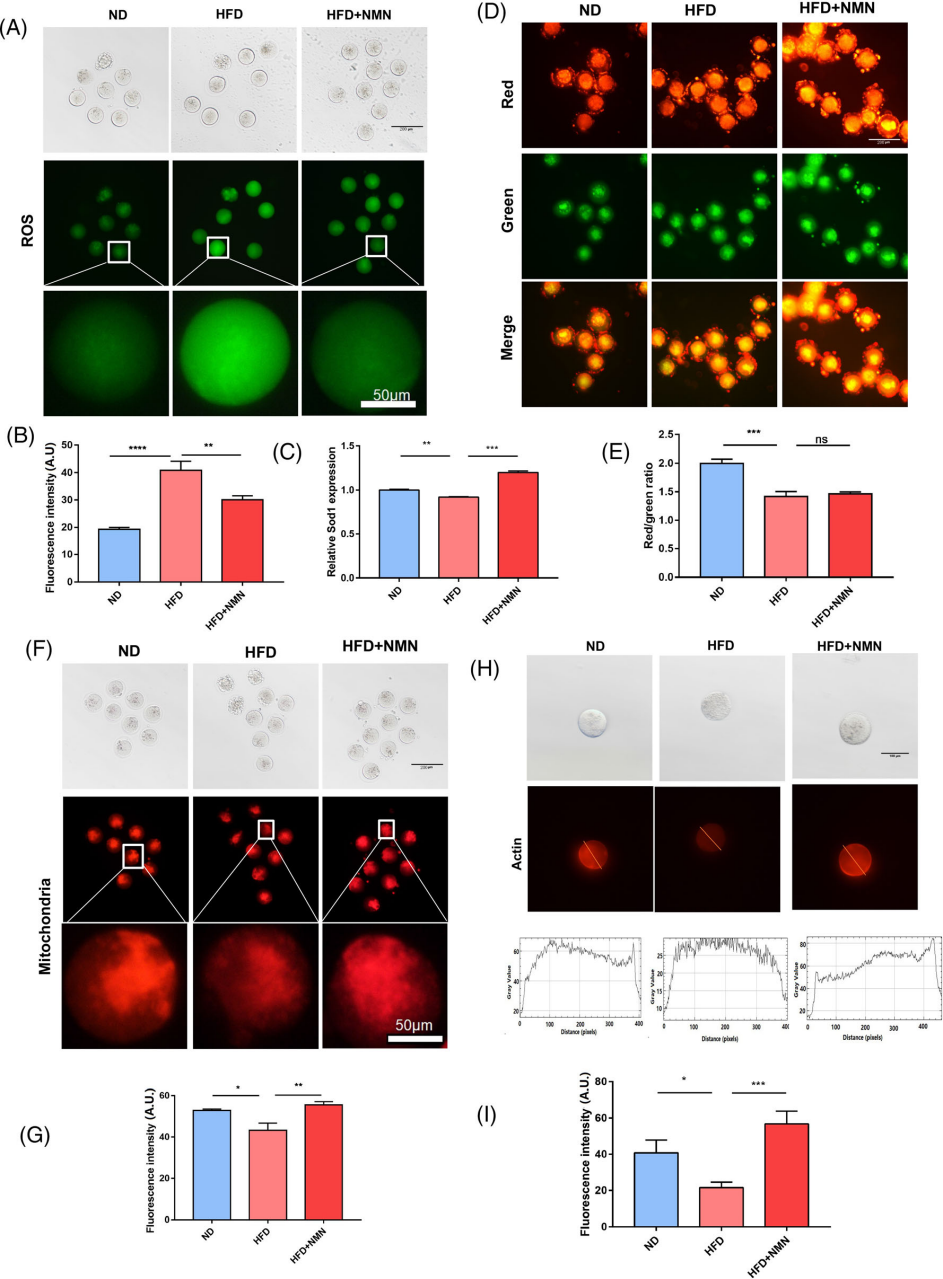
Effect of NMN on mitochondrial localization and function, ROS and the actin dynamics. (A) Representative images of ROS signals in ND, HFD and HFD + NMN mice oocytes. Scale bar, 200 μm, 50 μm. (B) The fluorescence intensity of ROS was quantified in ND, HFD and HFD + NMN mice oocytes. (C) mRNA level of Sod1 was examined by RT‐PCR in ND, HFD and HFD + NMN mice ovaries. (D) Mitochondrial membrane potential (ΔΨm) was assessed by JC‐1 staining in ND, HFD and HFD + NMN mice oocytes (red, high ΔΨm; green, low ΔΨm). Scale bar, 200 μm. (E) The ratio of red to green fluorescence intensity was quantified in ND, HFD and HFD + NMN mice oocytes. (F) Representative images of mitochondrial distribution in oocytes collected from ND, HFD and HFD + NMN mice. Scale bar, 200 μm, 50 μm. (G) The fluorescence intensity of mitochondrial signals was quantified in ND, HFD and HFD + NMN mice oocytes. (H) Representative images of actin signals and fluorescence intensity profiling of actin filaments in ND, HFD and HFD + NMN mice oocytes. Scale bar, 100 μm. (I) The fluorescence intensity of actin filaments on the membrane was quantified in ND, HFD and HFD + NMN mice oocytes. *P < 0.05; **P < 0.01; ***P < 0.001; ****P < 0.0001

ROS is a by‐product of oxidative phosphorylation which is produced by the mitochondria. Excessive ROS is harmful to many components of the cell such as DNA, proteins and lipids. Abnormal management of ROS also impaired oocyte quality and early embryonic development. Therefore, we examined ROS level in the indicated oocytes because ROS accumulation happened in obese mice oocytes and DNA damage process.^17^, ^18^ In oocytes from HFD mice, ROS signal was significantly stronger than that in the ND and HFD + NMN groups (Figure 5A,B), indicating that NMN reduced high oocyte oxidative stress induced by obesity. In addition, the results of qRT‐PCR showed that NMN could rescued downregulated expression of anti‐oxidation SOD1 in HFD group (Figure 5C).

Given that mitochondria are the main target of ROS and they play important roles in oocyte maturation and quality control, we further examined mitochondria function in terms of mitochondrial localization and membrane potentials in MII oocytes of the three groups. We evaluated the mitochondrial membrane potential (ΔΨm) of the oocytes of three groups by JC‐1 staining. A red fluorescence represented the high membrane potential, whereas a green fluorescence represented the low membrane potential. The results showed that the ratio of red to green signals of ND group was the highest among the ND group, the HFD group and the NMN‐supplemented HFD group (Figure 5D,E). In addition, the administration of NMN rescued the distribution of mitochondria (Figure 5F,G).

Given that actin dynamics is the key index of the oocyte quality, we then performed immunofluorescence experiment on the MII oocytes to examine whether HFD exerts detrimental effect on actin dynamics to further decrease oocyte quality. The results showed that the actin fluorescence intensity at the plasma membrane of HFD oocytes was lower than that of the ND group, and the supplement of NMN could significantly increase the actin fluorescence intensity at the plasma membrane of the HFD oocytes (Figure 5H,I). These results indicated that NMN could improve oocytes quality partially by maintaining the integrity of cytoskeleton.

### 3.6 NMN restored lipid droplet distribution of HFD mice

**FIGURE 6:**
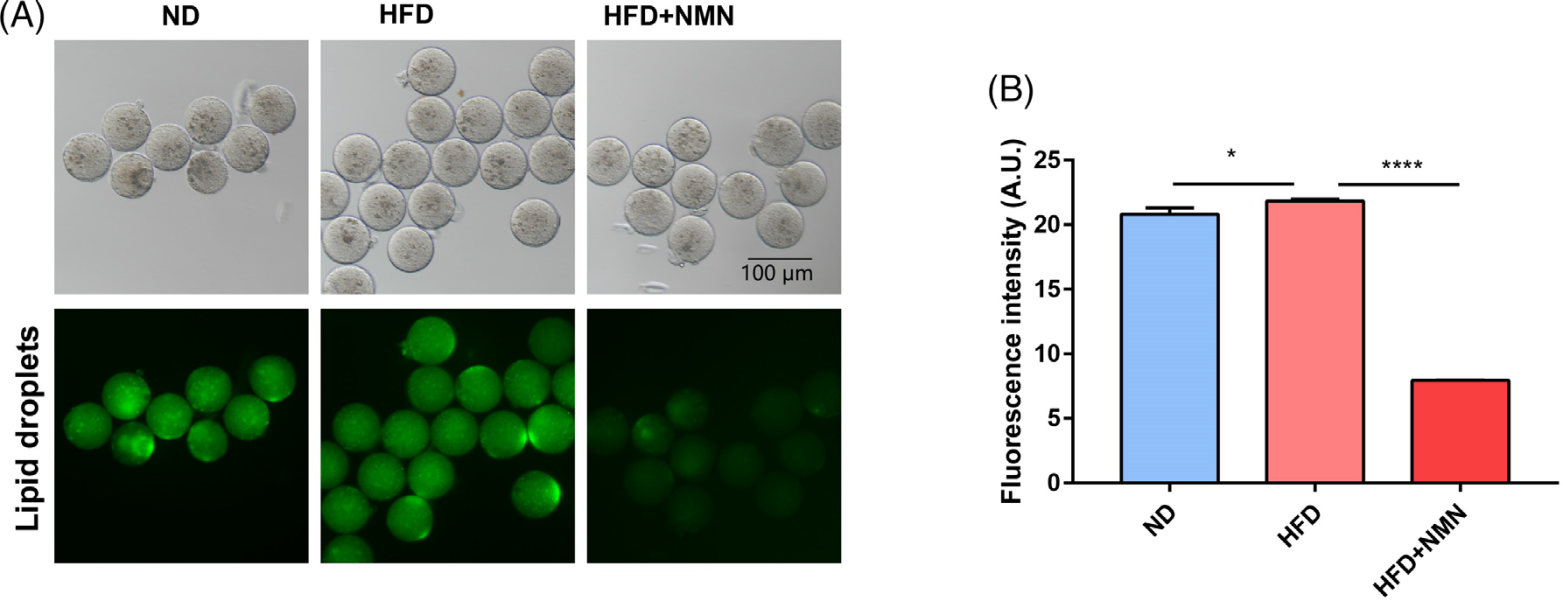
Effect of NMN on lipid droplets. (A) Representative images of lipid droplets in ND, HFD and HFD + NMN mice oocytes. Scale bar, 100 μm. (B) The fluorescence intensity of lipid droplets was quantified in ND, HFD and HFD + NMN mice oocytes. *P < 0.05; ****P < 0.0001

High fat diet could induce abnormal lipid droplet distribution in oocyte, which may be one of the reasons for the decline of oocyte quality. Therefore, we detected the lipid droplets in the indicated oocytes. Our results showed that the lipid droplet fluorescence intensity in HFD oocytes is slightly higher than that in ND oocytes, and the administration of NMN could reduce the fluorescence intensity of lipid droplets (Figure 6A,B).

### 3.7 NMN restored offspring weight of HFD mice

**FIGURE 7:**
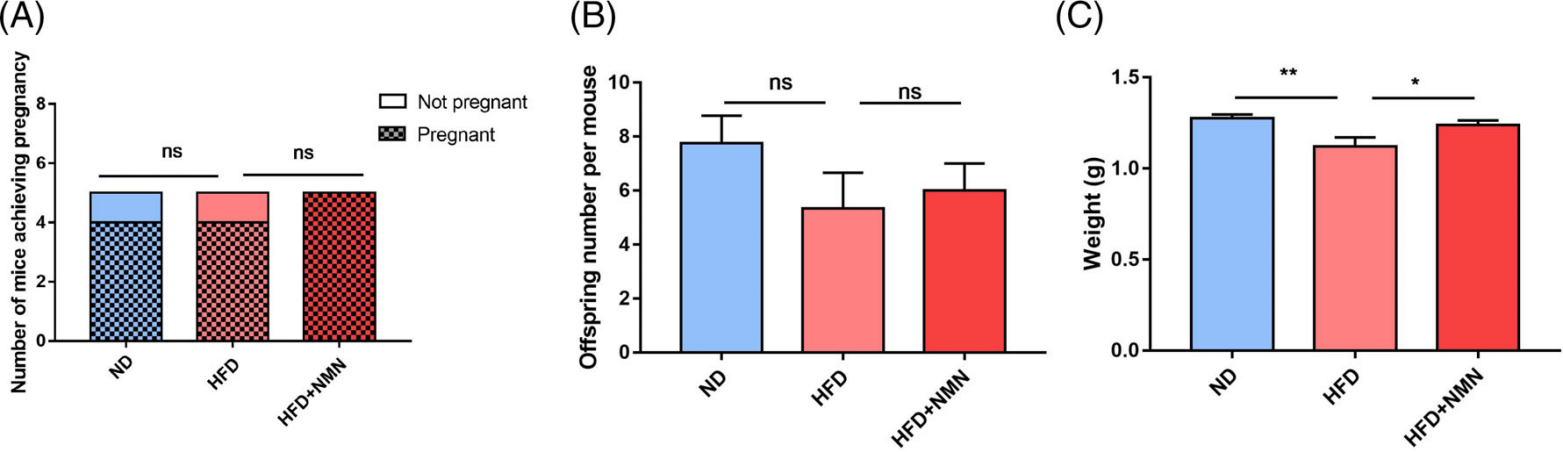
Effect of NMN on reproductive performance. (A) The pregnant rates of ND, HFD and HFD + NMN mice. (B) The pup number of ND, HFD and HFD + NMN mice. (C) The birth weight of pups of ND, HFD and HFD + NMN mice. *P < 0.05; **P < 0.01

We next sought to investigate the longer effect of NMN treatment on embryo development. The offspring numbers and weights in three groups were then assessed. There was no significant difference in the pregnancy probabilities of mice in three groups (Figure 7A). The female mice showed a similar litter size between three groups (Figure 7B). However, the birth weight of pups in the HFD group was significantly lower than that in the ND group, and the NMN supplement could rescue the reduced birth weight of pups caused by HFD (Figure 7C).

## 4 DISCUSSION

The obesity induced by high fat diet does harm to female fertility due to the possible damage on oocyte quality.^5^ Multiple drugs, such as melatonin, phycocyanin and resveratrol, have been developed to improve obese mice fertility.^19^, ^20^, ^21^ Recent studies reported that the administration of nicotinic acid (NA) and nicotinamide riboside (NR) could alleviate subfertility of obese mice by elevating NAD^+^ level.^12^, ^22^ NMN is a key NAD^+^ intermediate which has been shown effective in the improvement of oocyte quality in ageing mice.^14^, ^15^ However, the exact function of NMN on the obese mice fertility are not fully studied. In our study, we investigated the effect of NMN on ovaries and oocytes of HFD‐induced obese mice. Previous studies have shown that obesity induced by high fat diet could cause ovarian dysfunction and affect ovarian genes involved in folliculogenesis, thus reducing oocyte quality.^23^ We found that the ovary weight and ovarian organ index were lower in HFD group than that in ND group. We wondered if high fat diet and NMN administration would change the expressions of those genes such as Bmp4 and Lhx8, which were related to ovarian follicular development.^24^, ^25^ We found that the mRNA level of Bmp4 and Lhx8 were reduced in HFD group and NMN administration recovered the expression of these two genes. Above results revealed that the NMN administration reversed the abnormal ovary weight and the abnormal expression of folliculogenesis‐related genes including Lhx8 and Bmp4.

It has been reported that high fat diet leads to inflammation which plays important role in oocyte maturation and ovulation.^26^, ^27^ NMN has been shown to relieve LPS‐induced inflammation by suppressing pro‐inflammatory cytokine production.^28^ Our results showed that the expression of pro‐inflammatory genes including Adgre1, Ccl2, TNF‐α and Gal‐3 was upregulated but the expression of anti‐inflammatory genes including Clec10a and IL‐10 was downregulated in HFD group. Importantly, the NMN administration reversed their abnormal expression profile.

Normal spindle‐chromosome structure is essential for oocyte maturation and fertilization.^29^ The defective spindle‐chromosome complex (SCC) during meiosis can lead to aneuploidy, pregnancy loss and genetic diseases.^30^, ^31^ Studies have shown that the structure of the SCC in HFD mouse oocytes was disrupted.^5^, ^19^ Consistently, in our study, we found that HFD induced higher proportion of abnormal spindle‐chromosome structure and NMN could reduce this abnormal index.

Abnormality of ROS and mitochondria impaired oocyte quality and early embryonic development.^32^ Studies have indicated that ROS accumulates in oocytes of HFD mice, which accounts for DNA damage and apoptosis.^5^ The distribution pattern of mitochondrion has been considered as one of the important indicators for the oocyte cytoplasmic maturation because of its vital role for cell development.^33^ Previous studies on the effect of NMN on maternally aged oocytes have shown that NMN could eliminate the ROS level, recover mitochondrial function and reduce DNA damage.^14^ In order to assess the function of NMN on the quality of HFD oocytes, we then measured the ROS level, mitochondrial function and DNA damage of oocytes in ND group, HFD group and HFD + NMN group. We found that HFD induced a higher ROS level, damaged mitochondrial function and more DNA damage, and NMN treatment could recovered these indexes. These data indicated that NMN inhibited the ROS accumulation in oocytes of HFD mice which further improved oocyte quality of HFD mice. It was reported that the actin dynamics is involved in oocyte quality control and NMN could recover the abnormal actin dynamics in porcine oocytes.^34^ In line with this, we found that high fat diet disturbed the integrity of actin in oocytes and NMN could restore the actin dynamics in the HFD mice oocytes.

In our study, there was no significant difference on the pregnancy rate among the three groups. Moreover, previous study has shown that HFD mice ovulated smaller number of MII oocytes and produced less litter size.^20^ However, in our study, HFD mice could ovulated similar number of MII oocytes and produced a similar litter size compared with ND mice. Although there was no significant difference in litter size between the ND and HFD group, HFD mice showed a declined tendency in the litter size. The possible reason is that the genetic background of the mouse strains used in our study differed from that used in previous study.^20^ Furthermore, the mice age might also reduce the difference of the ovulation rate and litter size as these mice reached an older stage (more than 17 weeks) after the HFD treatment. Moreover, the little number of mice used in the experiment might affect the results. However, our results showed that the birth weight of pups in HFD group was lower than that in the control group, and NMN administration could help to recover their birth weight.

Altogether, we provided in vivo evidence to show that NMN has a capability to improve the quality of oocytes in HFD‐induced obese mice and revealed a potential mechanism that NMN could restore mitochondrial function, reduce ROS accumulation, DNA damage and lipid droplet distribution of HFD oocytes. Therefore, our work will provide a theoretical reference for improving the reproductive outcome of female obese people with the administration of NMN.

## AUTHOR CONTRIBUTIONS

Xiangpeng Dai conceived the project. Luyao Wang, Yurong Chen, Jiarui Wei, Fucheng Guo, Leyi Li, Zhen Han, Zhengzhu Wang, Haibo Zhu performed the experiment. Luyao Wang wrote the manuscript. Xiaoling Zhang, Ziyi Li and Xiangpeng Dai revised and edited the manuscript. All authors read and approved the final manuscript.

## CONFLICT OF INTEREST

All authors declare no conflict of interest.

## Supporting information


**Table S1** The raw Ct of quantitative real‐time PCR.

## Data Availability

The raw RT‐PCR data were listed in Supplementary Table 1. All other relevant data are available from the corresponding author upon reasonable request.
