# Supplementary material for: Administration of nicotinamide mononucleotide improves oocyte quality of obese mice
Source: Cell Prolif. 2022 Jul 10;55(11):e13303. doi: 10.1111/cpr.13303 (PMC9628229; doi:10.1111/cpr.13303)
Supplement: Supplementary file 1 — Table S1 The raw Ct of quantitative real‐time PCR. [file CPR-55-e13303-s001.doc]

Table S1. The raw Ct of quantitative real-time PCR.

| Sample Name | Target Name | Cт | Target Name | Cт |
| --- | --- | --- | --- | --- |
| CONTROL  CONTROL  CONTROL  HFD  HFD  HFD  HFD+NMN  HFD+NMN  HFD+NMN  CONTROL  CONTROL  CONTROL  HFD  HFD  HFD  HFD+NMN  HFD+NMN  HFD+NMN  CONTROL  CONTROL  CONTROL  HFD  HFD  HFD  HFD+NMN  HFD+NMN  HFD+NMN  CONTROL  CONTROL  CONTROL  HFD  HFD  HFD  HFD+NMN  HFD+NMN  HFD+NMN  CONTROL  CONTROL  CONTROL  HFD  HFD  HFD  HFD+NMN  HFD+NMN  HFD+NMN  CONTROL  CONTROL  CONTROL  HFD  HFD  HFD  HFD+NMN  HFD+NMN  HFD+NMN  CONTROL  CONTROL  CONTROL  HFD  HFD  HFD  HFD+NMN  HFD+NMN  HFD+NMN  CONTROL  CONTROL  CONTROL  HFD  HFD  HFD  HFD+NMN  HFD+NMN  HFD+NMN  CONTROL  CONTROL  CONTROL  HFD  HFD  HFD  HFD+NMN  HFD+NMN  HFD+NMN  CONTROL  CONTROL  CONTROL  HFD  HFD  HFD  HFD+NMN  HFD+NMN  HFD+NMN | actin  actin  actin  actin  actin  actin  actin  actin  actin  actin  actin  actin  actin  actin  actin  actin  actin  actin  actin  actin  actin  actin  actin  actin  actin  actin  actin  actin  actin  actin  actin  actin  actin  actin  actin  actin  actin  actin  actin  actin  actin  actin  actin  actin  actin  actin  actin  actin  actin  actin  actin  actin  actin  actin  actin  actin  actin  actin  actin  actin  actin  actin  actin  actin  actin  actin  actin  actin  actin  actin  actin  actin  actin  actin  actin  actin  actin  actin  actin  actin  actin  actin  actin  actin  actin  actin  actin  actin  actin  actin | 20.9301033  20.66985703  20.74748993  20.69108009  20.54539108  20.54818535  25.00903893  24.96547127  25.00974274  20.9301033  20.66985703  20.74748993  20.69108009  20.54539108  20.54818535  25.00903893  24.96547127  25.00974274  20.6360569  20.66440201  20.84043121  22.70008469  22.51082611  22.50606346  22.15511513  21.93872261  21.89933395  20.9301033  20.66985703  20.74748993  20.69108009  20.54539108  20.54818535  25.00903893  24.96547127  25.00974274  21.29084778  21.19316292  21.35019684  20.83257103  20.9484539  20.81115341  25.34188461  25.04379654  25.0347271  20.9301033  20.66985703  20.74748993  22.70008469  22.51082611  22.50606346  25.00903893  24.96547127  25.00974274  20.52690125  20.93949318  21.0890789  20.42749596  20.4791584  20.65139198  24.81494713  24.77510071  25.10759354  20.52690125  20.93949318  21.0890789  20.42749596  20.4791584  20.65139198  22.15511513  21.93872261  21.89933395  26.95648003  27.08719063  27.00591278  25.01862144  25.54434967  25.25078583  27.59875107  27.94919777  27.99819565  28.93961143  28.7191143  28.78742409  30.21962547  30.31160927  30.12799835  30.0158596  30.01890755  30.1569519 | Ccl2  Ccl2  Ccl2  Ccl2  Ccl2  Ccl2  Ccl2  Ccl2  Ccl2  TNF-α  TNF-α  TNF-α  TNF-α  TNF-α  TNF-α  TNF-α  TNF-α  TNF-α  Gal-3  Gal-3  Gal-3  Gal-3  Gal-3  Gal-3  Gal-3  Gal-3  Gal-3  Clec10a  Clec10a  Clec10a  Clec10a  Clec10a  Clec10a  Clec10a  Clec10a  Clec10a  IL-10  IL-10  IL-10  IL-10  IL-10  IL-10  IL-10  IL-10  IL-10  Adgre1  Adgre1  Adgre1  Adgre1  Adgre1  Adgre1  Adgre1  Adgre1  Adgre1  Lhx8  Lhx8  Lhx8  Lhx8  Lhx8  Lhx8  Lhx8  Lhx8  Lhx8  Bmp4  Bmp4  Bmp4  Bmp4  Bmp4  Bmp4  Bmp4  Bmp4  Bmp4  Bax  Bax  Bax  Bax  Bax  Bax  Bax  Bax  Bax  Sod1  Sod1  Sod1  Sod1  Sod1  Sod1  Sod1  Sod1  Sod1 | 30.77118111  30.9431057  30.885952  29.81022263  29.86857796  29.92901802  34.96045685  35.83914185  34.91135025  36.96992111  35.90832901  36.28421783  35.20183945  35.28574371  35.58841324  40.48819351  40.71735764  40.98564911  26.92719269  26.94390106  26.74662209  27.49869728  27.36699295  27.44502068  27.10911179  26.98048401  27.01676369  30.77042389  30.65255737  30.59070206  31.55547905  31.95201874  31.75444794  35.68238831  35.81044388  35.47366714  33.40929794  33.25301743  33.42272949  34.57764816  33.82773972  34.77436066  35.86444855  35.75953674  35.58164597  30.44476128  30.370924  30.23283958  31.59288597  31.38685989  31.07607841  34.94009781  34.44055176  34.41265488  28.90257645  28.90839195  28.9499321  32.80113602  32.65262222  32.82529449  32.96693802  33.00637817  32.66687393  30.7795105  30.8789978  30.53736305  31.41240883  31.09318161  31.36611557  32.08358383  32.28401947  32.34785843  32.91386795  32.66239166  32.44155502  30.95386314  30.73735619  30.9655323  33.65689468  33.84345245  33.71908951  27.93525505  27.95004463  27.90928268  29.46635628  29.46496964  29.43748283  28.94450569  28.92811012  28.87545013 |
